# Supplementary material for: Membrane computing simulation of sexually transmitted bacterial infections in hotspots of individuals with various risk behaviors
Source: Microbiol Spectr. 2024 Jan 10;12(2):e02728-23. doi: 10.1128/spectrum.02728-23 (PMC10845966; doi:10.1128/spectrum.02728-23)
Supplement: Supplemental tables — Tables S1 and S2. [file spectrum.02728-23-s0001.docx]

**SUPPLEMENTARY MATERIALS**

**Table S1. Mobility probability patterns (14 MPs, in columns) to visit sexual exchange hotspots (20 SEHs, in rows) corresponding to the various home residential areas (20 HRAs)**

| **MPs**  **SEHs** | **1** | **2** | **3** | **4** | **5** | **6** | **7** | **8** | **9** | **10** | **11** | **12** | **13** | **14** |
| --- | --- | --- | --- | --- | --- | --- | --- | --- | --- | --- | --- | --- | --- | --- |
| **1** |  |  | **0.75** |  |  |  |  |  |  |  |  |  |  |  |
| **2** |  |  | **0.25** | **0.5** |  |  |  |  |  |  |  |  |  |  |
| **3** |  |  |  | **0.25** |  |  |  |  |  |  |  |  |  |  |
| **4** |  |  |  | **0.25** | **0.25** |  |  |  |  |  |  |  |  |  |
| **5** |  |  |  |  | **0.5** | **0.5** |  |  |  |  |  |  |  |  |
| **6** |  |  |  |  | **0.25** | **0.5** | **0.5** |  |  |  |  |  |  |  |
| **7** |  |  |  |  |  |  | **0.5** | **0.1** |  |  |  |  |  |  |
| **8** |  |  |  |  |  |  |  | **0.4** |  |  |  |  |  |  |
| **9** |  |  |  |  |  |  |  | **0.4** |  |  |  |  |  |  |
| **10** | **0.9** | **0.1** |  |  |  |  |  | **0.1** |  |  |  |  |  |  |
| **11** | **0.1** | **0.9** |  |  |  |  |  |  | **0.1** |  |  |  |  |  |
| **12** |  |  |  |  |  |  |  |  | **0.4** |  |  |  |  |  |
| **13** |  |  |  |  |  |  |  |  | **0.4** |  |  |  |  |  |
| **14** |  |  |  |  |  |  |  |  | **0.1** | **0.5** |  |  |  |  |
| **15** |  |  |  |  |  |  |  |  |  | **0.5** | **0.5** | **0.25** |  |  |
| **16** |  |  |  |  |  |  |  |  |  |  | **0.5** | **0.5** |  |  |
| **17** |  |  |  |  |  |  |  |  |  |  |  | **0.25** | **0.25** |  |
| **18** |  |  |  |  |  |  |  |  |  |  |  |  | **0.25** |  |
| **19** |  |  |  |  |  |  |  |  |  |  |  |  | **0.5** | **0.25** |
| **20** |  |  |  |  |  |  |  |  |  |  |  |  |  | **0.75** |

**Table S2. Number of individuals from each of 20 home residential areas (HRAs) visiting 20 sexual hotspots accordingly with 14 mobility patterns (MPs)**

Example: In the first line of HRA01, MP03(Y) means that in HRA01 there are 5 MSF, 5 FSM, 60 MSM, and 30 FSF, all of them <35 years of age (Y), and all of them following the mobility pattern 3 (MP3)

| **HRA01** | | | | |
| --- | --- | --- | --- | --- |
|  | MSF-FSM | | MSM-FSF | |
|  | M | F | M | F |
| MP03(Y) | 5 | 5 | 60 | 30 |
| MP04(Y) | 5 | 5 | 40 | 20 |
| MP13(A) | 5 | 5 | 120 | 60 |
| MP14(A) | 5 | 5 | 80 | 40 |
| **HRA02** | | | | |
|  | MSF-FSM | | MSM-FSF | |
|  | M | F | M | F |
| MP03(Y) | 30 | 30 | 4 | 4 |
| MP04(Y) | 70 | 70 | 6 | 6 |
| MP13(A) | 80 | 80 | 6 | 6 |
| MP14(A) | 20 | 20 | 4 | 4 |
| **HRA03** | | | | |
|  | MSF-FSM | | MSM-FSF | |
|  | M | F | M | F |
| MP04(Y) | 50 | 50 | 10 | 10 |
| MP05(Y) | 50 | 50 | 10 | 10 |
| MP12(A) | 50 | 50 | 10 | 10 |
| MP13(A) | 50 | 50 | 10 | 10 |
| **HRA04** | | | | |
|  | MSF-FSM | | MSM-FSF | |
|  | M | F | M | F |
| MP04(Y) | 5 | 5 | 120 | 0 |
| MP05(Y) | 5 | 5 | 80 | 5 |
| MP12(A) | 5 | 5 | 120 | 0 |
| MP13(A) | 5 | 5 | 80 | 5 |
| **HRA05** | | | | |
|  | MSF-FSM | | MSM-FSF | |
|  | M | F | M | F |
| MP05(Y) | 5 | 5 | 0 | 150 |
| MP06(Y) | 5 | 5 | 5 | 50 |
| MP11(A) | 5 | 5 | 5 | 150 |
| MP12(A) | 5 | 5 | 0 | 50 |
| **HRA06** | | | | |
|  | MSF-FSM | | MSM-FSF | |
|  | M | F | M | F |
| MP05(Y) | 10 | 10 | 5 | 0 |
| MP06(Y) | 40 | 40 | 0 | 5 |
| MP11(A) | 40 | 40 | 5 | 0 |
| MP12(A) | 10 | 10 | 0 | 5 |
| **HRA07** | | | | |
|  | MSF-FSM | | MSM-FSF | |
|  | M | F | M | F |
| MP06(Y) | 100 | 100 | 10 | 10 |
| MP07(Y) | 100 | 100 | 10 | 10 |
| MP10(A) | 100 | 100 | 10 | 10 |
| MP11(A) | 100 | 100 | 10 | 10 |
| **HRA08** | | | | |
|  | MSF-FSM | | MSM-FSF | |
|  | M | F | M | F |
| MP07(Y) | 75 | 75 | 200 | 30 |
| MP08(Y) | 25 | 25 | 100 | 70 |
| MP09(A) | 75 | 75 | 200 | 40 |
| MP10(A) | 25 | 25 | 100 | 60 |
| **HRA09** | | | | |
|  | MSF-FSM | | MSM-FSF | |
|  | M | F | M | F |
| MP07(Y) | 50 | 50 | 50 | 200 |
| MP08(Y) | 50 | 50 | 50 | 100 |
| MP09(A) | 50 | 50 | 50 | 200 |
| MP10(A) | 50 | 50 | 50 | 100 |
| **HRA10** | | | | |
|  | MSF-FSM | | MSM-FSF | |
|  | M | F | M | F |
| MP03(Y) | 80 | 80 | 60 | 60 |
| MP05(Y) | 20 | 20 | 40 | 40 |
| MP12(A) | 80 | 80 | 60 | 60 |
| MP14(A) | 20 | 20 | 40 | 40 |
| **HRA11** | | | | |
|  | MSF-FSM | | MSM-FSF | |
|  | M | F | M | F |
| MP03(Y) | 140 | 60 | 140 | 50 |
| MP05(Y) | 60 | 40 | 60 | 50 |
| MP12(A) | 140 | 60 | 140 | 50 |
| MP14(A) | 60 | 40 | 60 | 50 |
| **HRA12** | | | | |
|  | MSF-FSM | | MSM-FSF | |
|  | M | F | M | F |
| MP04(Y) | 25 | 100 | 25 | 100 |
| MP06(Y) | 25 | 100 | 25 | 100 |
| MP11(A) | 25 | 100 | 25 | 100 |
| MP13(A) | 25 | 100 | 25 | 100 |
| **HRA13** | | | | |
|  | MSF-FSM | | MSM-FSF | |
|  | M | F | M | F |
| MP04(Y) | 25 | 25 | 10 | 10 |
| MP06(Y) | 75 | 75 | 10 | 10 |
| MP11(A) | 30 | 30 | 10 | 10 |
| MP13(A) | 70 | 70 | 10 | 10 |
| **HRA14** | | | | |
|  | MSF-FSM | | MSM-FSF | |
|  | M | F | M | F |
| MP05(Y) | 55 | 60 | 15 | 5 |
| MP07(Y) | 45 | 40 | 5 | 15 |
| MP10(A) | 45 | 45 | 15 | 15 |
| MP12(A) | 55 | 55 | 5 | 5 |
| **HRA15** | | | | |
|  | MSF-FSM | | MSM-FSF | |
|  | M | F | M | F |
| MP05(Y) | 5 | 5 | 180 | 5 |
| MP06(Y) | 5 | 5 | 20 | 0 |
| MP10(A) | 5 | 5 | 180 | 5 |
| MP12(A) | 5 | 5 | 20 | 0 |
| **HRA16** | | | | |
|  | MSF-FSM | | MSM-FSF | |
|  | M | F | M | F |
| MP04(Y) | 5 | 5 | 5 | 120 |
| MP05(Y) | 5 | 5 | 0 | 80 |
| MP12(A) | 5 | 5 | 5 | 120 |
| MP13(A) | 5 | 5 | 0 | 80 |
| **HRA17** | | | | |
|  | MSF-FSM | | MSM-FSF | |
|  | M | F | M | F |
| MP04(Y) | 5 | 5 | 100 | 5 |
| MP05(Y) | 5 | 5 | 100 | 0 |
| MP12(A) | 5 | 5 | 100 | 5 |
| MP13(A) | 5 | 5 | 100 | 0 |
| **HRA18** | | | | |
|  | MSF-FSM | | MSM-FSF | |
|  | M | F | M | F |
| MP05(Y) | 5 | 5 | 5 | 110 |
| MP06(Y) | 5 | 5 | 0 | 90 |
| MP11(A) | 5 | 5 | 5 | 110 |
| MP12(A) | 5 | 5 | 0 | 90 |
| **HRA19** | | | | |
|  | MSF-FSM | | MSM-FSF | |
|  | M | F | M | F |
| MP04(Y) | 0 | 0 | 200 | 100 |
| MP05(Y) | 100 | 100 | 100 | 200 |
| MP06(Y) | 100 | 100 | 100 | 100 |
| MP11(A) | 100 | 100 | 120 | 80 |
| MP12(A) | 100 | 100 | 120 | 80 |
| MP13(A) | 0 | 0 | 60 | 140 |
| **HRA20** | | | | |
|  | MSF-FSM | | MSM-FSF | |
|  | M | F | M | F |
| MP03(Y) | 300 | 280 | 0 | 0 |
| MP05(Y) | 130 | 100 | 20 | 15 |
| MP07(Y) | 100 | 100 | 20 | 25 |
| MP10(A) | 300 | 280 | 20 | 45 |
| MP12(A) | 130 | 100 | 20 | 45 |
| MP14(A) | 100 | 100 | 0 | 0 |
